# Editorial: Exploring the potential of nanobiomaterials in biomedical engineering: assessing biocompatibility, toxicity, and future prospects

**DOI:** 10.3389/fchem.2024.1461483

**Published:** 2024-10-23

**Authors:** Narsimha Mamidi, Ebrahim Mostafavi, Murali M. Yallapu

**Affiliations:** ^1^ Wisconsin Center for NanoBioSystems, Pharmaceutical Sciences Division, School of Pharmacy, University of Wisconsin-Madison, Madison, WI, United States; ^2^ Department of Medicine, Stanford University School of Medicine, Stanford, CA, United States; ^3^ Department of Immunology and Microbiology, Medicine and Oncology ISU, School of Medicine, The University of Texas Rio Grande Valley, McAllen, TX, United States

**Keywords:** nanobiomaterials, biocompatibility, toxicity, drug delivery, biomedical applications, tissue engineering

Nanomedicine is poised for a groundbreaking shift that is driven by nanobiomaterials (NBMs). (Tang et al., 2024; Mamidi et al., 2022a; Zhang et al., 2024). These innovative materials bridge the gap between nanotechnology and biomaterials, offering unique properties that can revolutionize healthcare. NBMs are engineered at the nanoscale, typically ranging from 1 to 100 nm, which is comparable to many biological structures in the human body. (Orash Mahmoud Salehi et al., 2022). This scale allows NBMs to interact with cells and tissues on a fundamental level, mimicking their natural structures and functions. The excitement around NBMs is driven by their potential advantages. Their high-loading capacity allows them to act as efficient carriers for drugs, genes, or other therapeutic agents. (Mamidi et al., 2020; Mamidi et al., 2022b; Mamidi and Delgadillo, 2021; Mamidi et al., 2023). Additionally, NBMs can be engineered with improved mechanical properties, making them ideal for use as implants or scaffolds that support tissue regeneration. They can be customized with specific optical, electrical, and magnetic functionalities, paving the way for advancements in the fields of diagnostics, biosensing, and targeted therapies. (Mamidi et al.; Mamidi and Jesús Fernando Flores Otero, 2023). However, the promise of NBMs is accompanied by a crucial caveat: safety. Due to their nanoscale size and unique physicochemical properties, NBMs may interact with the body in unpredictable ways. They can cross biological barriers and accumulate in unintended locations, potentially causing adverse effects. Moreover, their interaction with cells and tissues needs careful evaluation to ensure they do not trigger unwanted immune responses, oxidative stress, or genetic damage.

Recognizing these potential risks, researchers are diligently assessing the biocompatibility and toxicity of NBMs through comprehensive *in vitro* and *in vivo* testing. The current research aims to understand how NBMs interact with living systems, focusing on various factors such as size, morphology, shape, and surface chemistry, which significantly influence their behavior in the human body. Acquiring such knowledge is critical for designing safe and effective NBMs for clinical applications. Current research trends emphasize addressing the safety concerns of NBMs. Scientists are striving to establish standardized testing protocols and certified standards to ensure consistent and reliable evaluation of NBMs. By thoroughly understanding the potential risks, researchers can develop strategies to mitigate them, thus paving the way for the safe and ethical use of NBMs in medicine.

**FIGURE 1:**
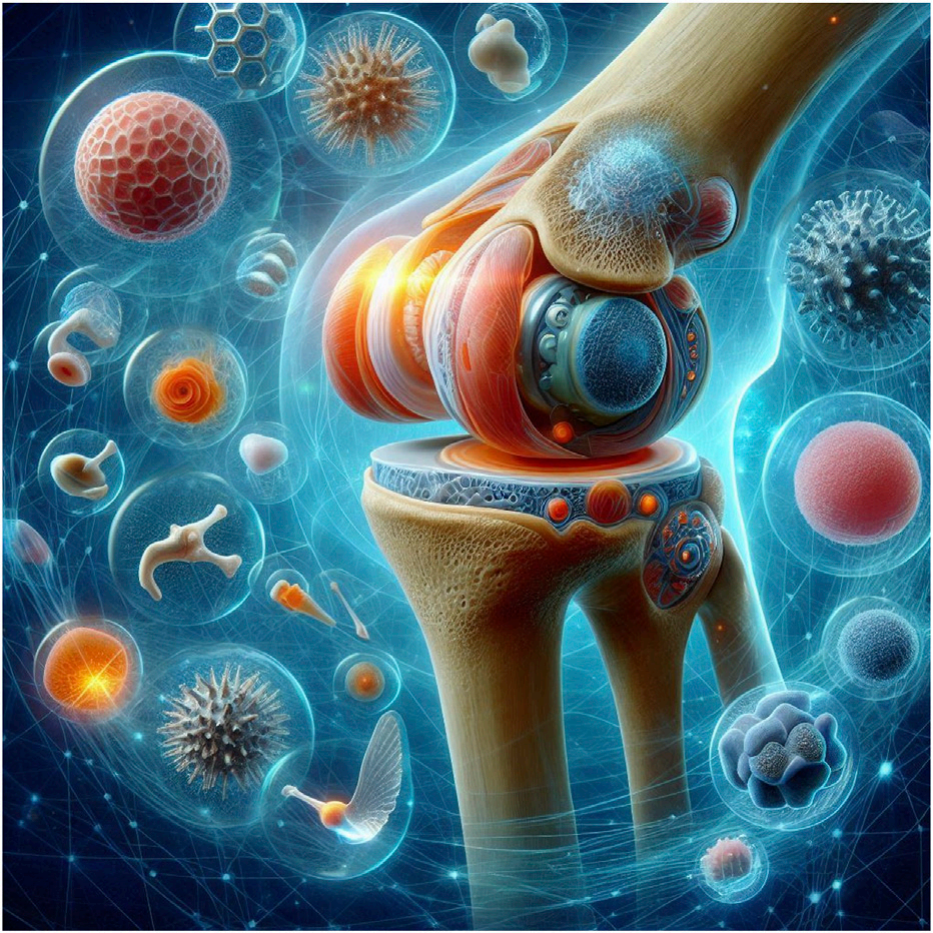
A cartoon depicting a nanobiomaterial-based knee implant designed for improved functionality and patient outcomes.

The future of NBMs holds immense promise. By leveraging the capabilities of nanotechnology and emulating the intricate structures of living tissues, NBMs offer exciting possibilities for diverse biomedical applications (Figure 1). These include targeted drug delivery systems to advanced implants to improve human health. However, this progress hinges on a commitment to rigorous safety assessments and a deep understanding of how NBMs interact with biological systems. Through ongoing research and development, NBMs can transform how we diagnose, treat, and prevent diseases, heralding a new era of personalized and targeted medicine.

This Research Topic explores the vital interactions between NBMs and cells/tissues, focusing on how factors such as size, morphology, shape, and surface chemistry of NBM influence toxicity, biocompatibility, and immunogenicity. By elucidating these structure-function relationships, we aim to facilitate the creation of innovative NBMs with tailored properties for diverse biomedical applications. This Research Topic includes three research papers and two review articles exploring recent advances in the design, preparation, and biocompatibility assessment of NBMs.

Guedri et al. investigated the potential of using alumina-ferrite (Al_2_O_3_-Fe_3_O_4_) hybrid nanoparticles to improve thermal systems in various fields, including biomedicine, electronics, and mechanical engineering. (Guedri et al.). Their study focused on the interaction of these nanoparticles with blood over a 3D surface, considering factors like nonlinear thermal radiation, stretching, velocity slippage, and a magnetic field. A mathematical model was developed using nanofluid properties and similarity rules. Numerical simulations were performed to analyze the behavior of the nanoparticles within the blood. The results showed that a strong magnetic field effectively controlled the motion of the nanoparticles, while surface stretching increased fluid movement. Thermal radiation was found to enhance the thermal properties of both Al_2_O_3_-Fe_3_O_4_/blood and Al_2_O_3_/blood. (Guedri et al.).

In a review of biomedical applications for iron sulfide-based nanozymes (ISNs), Shan et al. highlighted the growing interest in nanozymes due to their stability, ease of preparation, and tunability. (Shan et al.). They focused on ISNs, a widely studied nanomaterial with enzyme-mimicking properties, outlining their potential in various biomedical applications. The review explores the classification and catalytic mechanisms of ISNs, followed by a detailed examination of their use in biosensors, tumor therapy, antibacterial therapy, and other areas, underlining their promise for improving human health.

In another study, Puri et al. explored the green synthesis of selenium nanoparticles (SeNPs) using Terminalia arjuna bark extract. (Puri et al.). The abundant phenolics, flavonoids, and tannins in the extract acted as capping and stabilizing agents, enabling the formation of stable, negatively charged, and spherical TA-SeNPs. These biogenic SeNPs exhibited excellent antioxidant, antibacterial, and anticancer activities, making them promising candidates for biomedical applications. Notably, TA-SeNP-incorporated gel displayed desirable properties for topical use. This study highlights the potential of biogenic SeNPs for the safe and sustainable development of nanomedicines.

Roma and Hegde reviewed recent advancements in graphene and its derivatives for dental applications. (Roma and Hegde). Highlighting their unique properties like biocompatibility and antibacterial activity, the authors discuss synthesis methods, material characteristics, and various dental uses of graphene-based materials. The review concludes by exploring the challenges and future potential of these nanomaterials in dentistry, aiming to stimulate further research.

A study by Dar et al. investigated the effects of various nutrient sources on rice growth and yield. (Puri et al., 2024). Eight practices were evaluated, including a recommended fertilizer dose (RFD), RFD with a silicon supplement, and organic manure (FYM). The treatment with a basal application of Vigore (a commercial product) followed by a spray at the panicle initiation (PI) stage (N_3_) resulted in the highest plant height, number of tillers, panicle density, panicle weight, and grain yield. This approach yielded 22%–25% more grain than RFD with FYM or RFD alone. The study suggests that silicon and targeted application timing can significantly improve rice yield.

Altogether, the articles presented in this Research Topic highlight significant progress in the field of nanobiomaterials. We extend our sincere gratitude to all contributing authors for sharing their valuable insights and solutions. Special thanks go to the editors and reviewers whose expertise significantly enhanced the quality of the papers. Despite these advancements, challenges remain. Future success hinges on rigorous safety assessments and a deeper understanding of NBM interactions with biological systems. Continued research and development hold the potential for NBMs to revolutionize disease diagnosis, treatment, and prevention, paving the way for personalized and targeted medicine. We trust that the articles in this Research Topic will be both informative and inspiring, particularly for young scholars eager to contribute to the future of NBM research.
